# Long-COVID syndrome: physical–mental interplay in the spotlight

**DOI:** 10.1007/s10787-023-01174-4

**Published:** 2023-03-09

**Authors:** Carolin Thurner, Andreas Stengel

**Affiliations:** 1 Department of Psychosomatic Medicine and Psychotherapy, University Hospital Tübingen, Tübingen, Germany; 2 Charité Center for Internal Medicine and Dermatology, Medical Clinic for Psychosomatic Medicine, Charité Universitätsmedizin Berlin, Corporate Member of Freie Universität Berlin, Humboldt-Universität Zu Berlin and Berlin Institute of Health, Berlin, Germany

**Keywords:** Biopsychosocial model, Coronavirus, Multidisciplinary, Multi-professional, SARS-CoV-2, Treatment, Understanding

## Abstract

Patients suffering from Long-COVID syndrome experience a variety of different symptoms on a physical, but also on a psychological and social level. Previous psychiatric conditions such as depression and anxiety have been identified as separate risk factors for developing Long-COVID syndrome. This suggests a complex interplay of different physical and mental factors rather than a simple cause–effect relationship of a specific biological pathogenic process. The biopsychosocial model provides a foundation for understanding these interactions and integrating them into a broader perspective of the patient suffering from the disease instead of the individual symptoms, pointing towards the need of treatment options on a psychological as well as social level besides biological targets. This leads to our conclusion, that the biopsychosocial model should be the underlying philosophy of understanding, diagnosing and treating patients suffering from Long-COVID syndrome, moving away from the strictly biomedical understanding suspected by many patients, treaters and the media while also reducing the stigma still associated with the suggestion of a physical–mental interplay.

## Introduction

According to the NICE-guidelines, Long-COVID syndrome is defined as “signs and symptoms that develop during or following an infection consistent with COVID-19 and which continue for more than four weeks and are not explained by an alternative diagnosis” (Sivan and Taylor 2020). The prevalence varies greatly between different reports, depending on the patient population, selection process, time after infection and the method of recording symptoms. The most common symptoms include fatigue (Joli et al. 2022), dyspnea and reduced cognitive and physical performance (Lopez-Leon et al. 2021). For many patients, these symptoms recede spontaneously, while others report persisting symptoms over months or even years (Anaya et al. 2021; Lund et al. 2021). The clinical picture is usually complex, lacking specific laboratory values and leading to guidelines recommending an interdisciplinary approach, taking into account the whole person and continuity of treatment (Koczulla et al. 2022). While there are continuing advances in research, the pathogenesis is not clear, multifactorial and probably not the same for all patients (Koczulla et al. 2022). Apart from age, risk factors for developing Long-COVID syndrome are pre-existing conditions, especially hypertension, obesity, psychiatric conditions and immunosuppression (Crook et al. 2021). Interestingly, a French study of 26,823 adults during the COVID-19 pandemic found an association of self-reported COVID-19 infection with persistent physical symptoms, whereas laboratory-confirmed COVID-19 infection was associated only with anosmia (Matta et al. 2022).

Many different factors stress the importance of including a perspective focusing on physical–mental interplay in the diagnostic and treatment process. First, Long-COVID syndrome leads to mental stress and symptoms such as depression, anxiety (Silva Andrade et al. 2021) and in some cases post-traumatic stress disorder, especially if severe dyspnea was experienced during the infection (Harenwall et al. 2022). Second, pre-existing psychiatric illnesses are a separate risk factor of developing Long-COVID syndrome (Yong 2021), emphasizing a close connection between pathogenic mechanisms. Third, the COVID-19 pandemic with a fear of infection, social distancing, a rise in unemployment and growing uncertainty sparked a clear rise in both stress and psychiatric diseases worldwide (Chen et al. 2021), leading some authors to speculate about a new diagnostic category for specific mental disorders resulting from the COVID-19 pandemic in the future (Heitzman 2020). Fourth, the resemblance of Long-COVID syndrome to other somatoform disorders such as irritable bowel syndrome are obvious, with both disorders characterized by somatic pathogenetic alterations in close interaction with psychological factors (Koczulla et al. 2022; Chey et al. 2015). Lastly, symptoms of fatigue as well as decreased cognitive and physical performance are key diagnostic criteria for depression.

Especially with a rising complexity of a disease such as in the case of Long-COVID syndrome, the biopsychosocial model can play a central role in helping to understand diseases and as a guideline for the development of treatment plans.

## The biopsychosocial model

One of the pillars of a modern comprehensive understanding of diseases is the biopsychosocial model introduced by Engel (1978). Engel proposed a counterpoint to the strictly biomedical model of illness, which is mostly concerned with directly measurable, structural pathologies and is the primary model physicians are trained in and patients come to expect in an increasingly technical world. This, however, neglects to include the vast amount of knowledge about human behavior and other psychological and social influences on the development, course and subjective perception of symptoms. Engel accuses this biomedical model of dualism by strictly separating the mind and body, the mental from the physical, and of reductionism by trying to understand an extremely complex entity such as life itself by analysis of its component parts and explaining them in the language of physics and (bio-)chemistry. Engel did not deny the obvious and amazing advances in biomedical research and treatments, which have progressed even farther since the first introduction of his model. However, this implies a promise of a complete understanding of all diseases and availability of treatment options which until today has not been achieved – even if this has become the expectation of many patients seeking care. This has unfortunately shifted perspective from the comprehensive and individual clinical assessment to various laboratory and diagnostic procedures, which stand in their own right in some cases and are overemphasized in others. In many cases, this leads to a discrepancy between the subjective symptoms and limitations the patients experience in day-to-day life and a lack of measurable biomedical markers, which is extremely frustrating for both physician and patient, especially if this biomedical model dominates both the perception of illnesses and the communication between physician and patient as well as in society in general. Today, this is massively supported by public reporting, which frequently presents visually impressive, sometimes pseudo-scientific diagnostic and treatment methods as highly promising standards of medical care and dismisses the physical–mental interplay, increasing the already existing stigma. This model inevitably leads to the exclusion of the main character - the patient - from their own disease. In some diseases, if the correlations between biomedical alterations and resulting symptoms are relatively linear, appropriate treatment options are available and the existing psychosocial coping strategies outside the medical field are effective enough, this model seems to be sufficient. However, with a rising complexity and especially chronification of illnesses, this approach falls short in both explaining symptoms and treating patients.

This crisis inspired Engel to the introduction of his biopsychosocial model, aiming to be more inclusive and provide a framework to conceptualize all levels of health and disease, “from subatomic particles through molecules, cells, tissues, organs, organ systems, the person, the family, the community, the culture, and ultimately the biosphere” (Engel 1978) where every system is relatively autonomous, but interconnected with every other system through feedback arrangements. Instead of linear causality, it expects reciprocal causal effects, which can carry disturbances from one system to another. In this model, overall health and illness are dependent on the relative intactness and functioning of each component, communication and intra- and intersystemic harmony. In addition, every change becomes part of the history of its system, underlining the dynamic quality. Health is not a single state, which is to be achieved, but the quality of overall harmony, which can vary before and after the disturbance. This change of perspective has huge implications both on the perception of illness and of the communication with the patient. Instead of reducing the patient to just a few specific symptoms or measurable parameters and treatment by a purely biomedical intervention, they are understood on a more complete basis, and the biomedical intervention becomes one part of a comprehensive treatment plan on many different levels.

While this model finds its most prominent appliance in somatoform disorders (Kreipe 2006; Henningsen 2018) and psychiatric diseases (Papadimitriou 2017), it is by no means limited to those. From patients with psychiatric disorders being more likely to e.g. develop and have a higher mortality rate for cardiovascular disorders (Hare et al. 2014) and cancer (Pinquart and Duberstein 2010) to psychosocial factors being an important pathogenic factor in the development and outcome of Crohn’s disease (Ringel and Drossman 2001) and rheumatological illnesses, increasing fatigue in patients suffering from lupus erythematosus (Aberer 2010), there is a wide range of evidence supporting this model. In addition, it has been shown that biographical trauma and stress substantially influence pain perception and processing (Tesarz et al. 2018).

## Applications of the biopsychosocial model to the understanding of Long-COVID syndrome

As the possible biological factors associated with Long-COVID syndrome are discussed in the other articles of this special issue, we will not further embark on those, but concentrate on psychological and social contributors as well as interactions between the different systems.

Apart from the stress on the medical system with doctors and nurses working to the limit of their capacities to treat patients, the coronavirus pandemic had huge effects on society and individuals on many different levels, leading to an increase in mental health problems in the general population, but especially in patients infected with SARS-CoV-2  (Hossain et al. 2020). Although most of those seemed to recover quickly (Manchia et al. 2022), patients suffering from Long-COVID syndrome had an increase in mental health problems such as depression and anxiety (Silva Andrade et al. 2021). This could be argued to simply be a result of the limitations and restrictions due to the disease. However, pre-existing psychiatric conditions were identified as separate risk factors for a prolonged recovery after COVID-19 (Crook et al. 2021) and developing Long-COVID syndrome (Yong 2021), suggesting a much more complex interaction. Patients with mental health problems usually experienced some form of biographical trauma or stress, resulting in inner conflicts and a reduced ability to cope with internal and external stressors. Due to the nature of these conflicts and personality structure, these are at times projected into the body and experienced as somatic symptoms such as fatigue, gastrointestinal problems, pain or cardiac reactions (Mentzos 2017). These symptoms, while very real in their restrictions to the patients’ lives and associated with actual somatic symptoms (such as diarrhea, tachycardia or heightened muscle tension as a correlate for pain) are not the result of structural damage of the respective organs, but of dysregulation due to imbalances on a psycho-neurological scale (e.g., “gut-brain-axis”), which have reciprocal effects in both directions and are linked to a “diminished capacity to consciously experience and differentiate affects and express them in an adequate or healthy way” (Waller and Scheidt 2006). Moreover, physical and mental health problems influence each other significantly. It has been shown for example, that depression and pain perception have a bidirectional influence, increasing each other, even holding true for acute pain due to injury or trauma (Michaelides and Zis 2019).

So what could that mean for our understanding of Long-COVID syndrome? It has been well established, that apart from physical symptoms, Long-COVID syndrome is associated with psychological and social aspects, both as separate risk factors and symptoms of the disease. This is not only a finding consistently established in various studies mentioned above, but easily understandable from a biopsychosocial perspective, as many examples show. One of the main symptoms in patients suffering from Long-COVID syndrome is physical and mental fatigue. This often leads to the development of depressive symptoms, including avolition and a general feeling of hopelessness and heaviness, increasing fatigue. In addition, patients are often afraid of the sometimes very intense “crashes” of post-exertional malaise, leading to a reduction of physical and mental exercise, which in turn further decreases the respective abilities and condition. Reduced social participation can increase this dynamic, as social isolation reduces demands and progresses depressive symptoms. With a reduced capacity to cope with the difficulties of the disease, patients with pre-existing clinical (or subclinical) psychiatric diagnoses are much more vulnerable to these processes. This could lead to symptoms persisting much longer than any single biological cause could induce. It is important to understand that these mechanisms are neither a sideline effect nor the fault of the patient but an integral part of the pathogenic process.

**Fig. 1:**
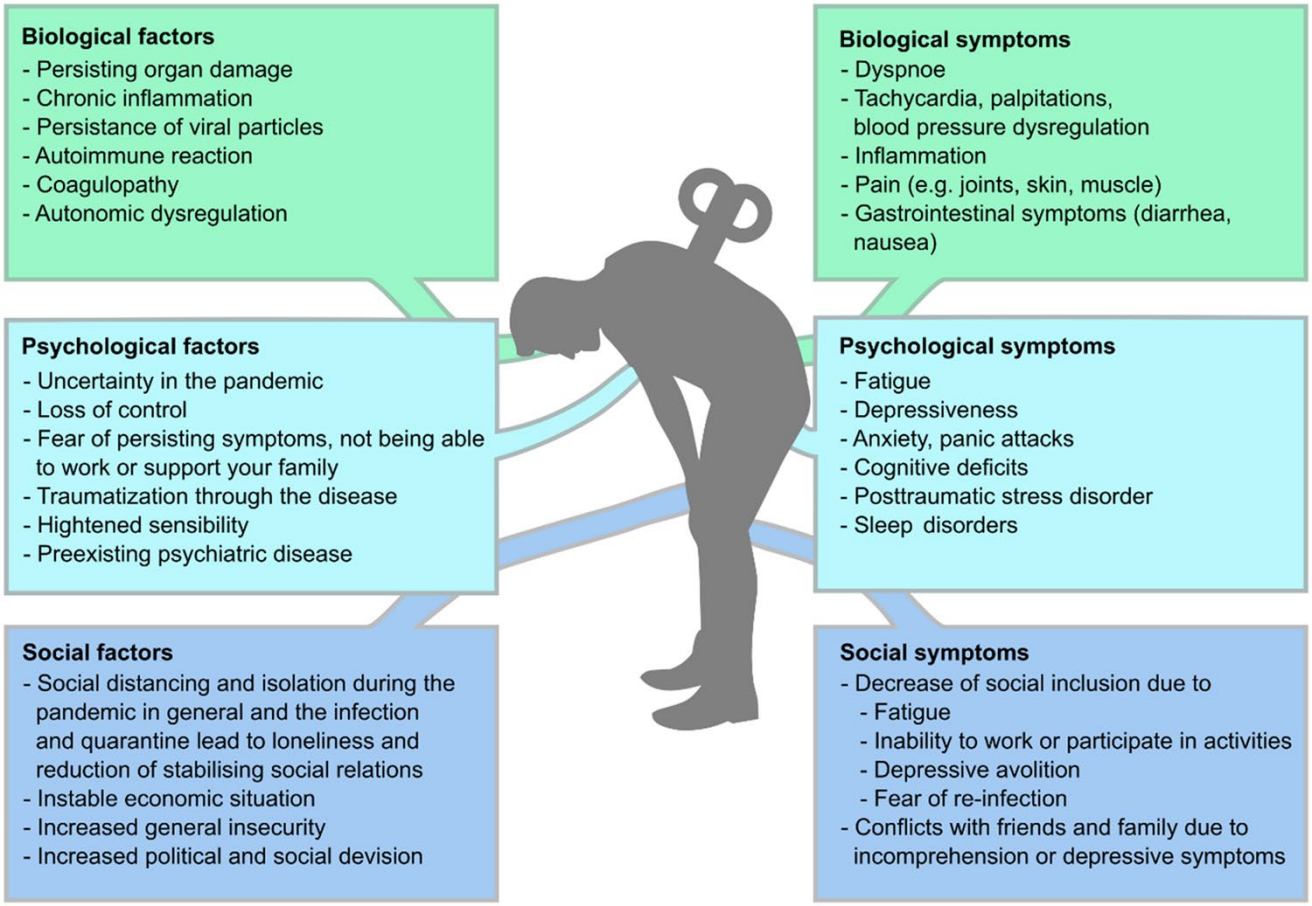
Contributing biological, psychological and social factors and resulting symptoms in patients with Long-COVID syndrome

In addition, attempts to establish either a single biological cause or treatment option have so far failed, pointing towards the insufficiency of this approach. This leads to our suggestion not to focus merely on Long-COVID syndrome but rather on the patients suffering from Long-COVID syndrome, shifting our perspective to include all biopsychosocial aspects of these patients instead of a list of symptoms to be eliminated (Fig. 1). We, therefore, need to broaden our understanding of how these aspects interact with each other.

## Consequences for treatment plans

Even before the symptoms of Long-COVID syndrome became widely known and focus of further research, it was argued that the biopsychosocial model should be the underpinning philosophy in rehabilitation for patients recovering from COVID-19 (Wainwright and Low 2020). The authors recognized patients’ needs not only for physical treatment, but psychological and social support as well, while the focus on different aspects may (and should) vary during the course of treatment and between individuals. There is evidence, that post-traumatic stress disorder in patients with Long-COVID syndrome was directly associated with increased fatigue and breathlessness and that improvements in fatigue after rehabilitation were associated with improvements in post-traumatic stress disorder (Harenwall et al. 2022), which led the authors to emphasize the need to apply the biopsychosocial approach in Long-COVID rehabilitation and the integration of psychotherapy as treatment for post-traumatic stress disorder as a priority in treatment. This is mirrored in official consensus statements and guidelines for Long-COVID rehabilitation and treatment, such as from Stanford Hall (Barker-Davies et al. 2020) and the German association of medical societies (Koczulla et al. 2022) explicitly including psychological teams in the rehabilitation and treatment process.

This biopsychosocial model has already been successfully implemented in a digital Long-COVID rehabilitation program consisting of an interdisciplinary team of health professionals led by a clinical psychologist and including a physiotherapist, occupational therapist, dietitian, speech and language therapist, assistant psychologist and personal support navigator, which has greatly improved symptoms (Harenwall et al. 2021).

Not surprisingly, there is evidence suggesting a benefit of antidepressive medication in treating depressive symptoms in patients suffering from Long-COVID syndrome (Fenton and Lee 2023; Mazza et al. 2022). Other mental illnesses - if present - should be treated according to the respective national and international guidelines. However, there is currently no general evidence for psychiatric pharmacotherapy for all Long-COVID cases, therefor the indication, as with all treatment components, should be assessed on an individual basis.

**Fig. 2:**
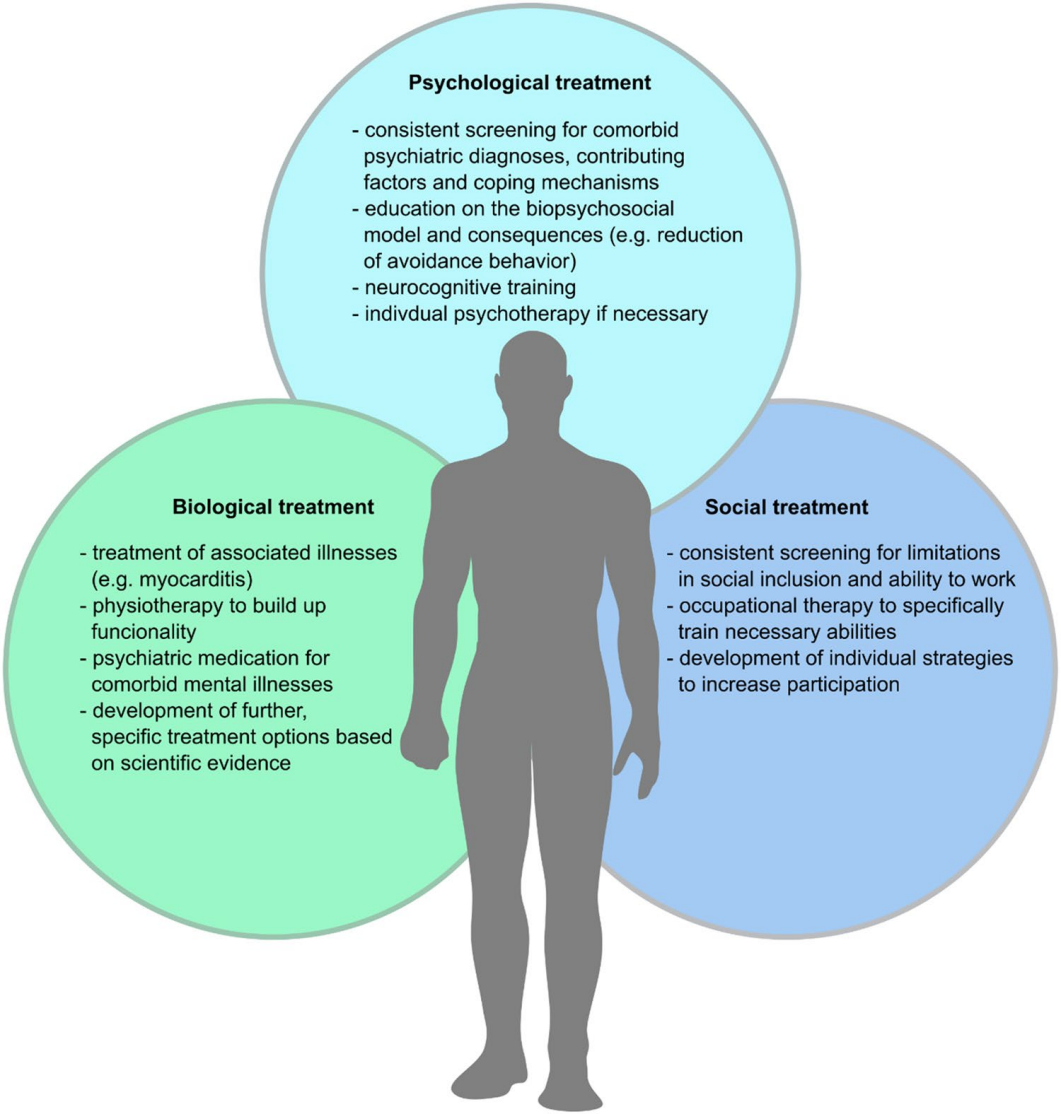
Treatment plans including the biopsychosocial model

Based on this, we suggest the further development of comprehensive treatment plans, including all aspects of the patient. Apart from treating known diseases on a biological level (e.g., myocarditis) if present, biological treatment should focus on physiotherapy and other rehabilitation methods. This should be augmented with a consistent screening for psychiatric diagnoses as well as basic psychological interventions, such as education on the biopsychosocial model and its implications, motivating the patient to reduce avoidance behavior and limiting anxiety. Neurocognitive training and individual psychotherapy as well as psychiatric pharmacotherapy should be applied where necessary. On a social scale, occupational therapy could be a tool to specifically train necessary abilities to enable participation in both social and work life (Fig. 2).

## Conclusion

Especially since the current public and medical understanding as well as scientific practice often encourages the belief in unidirectional cause–effect relationships with a great stigmatization of psychiatric or psychosomatic disorders as well as physical–mental interaction in general, it should be our goal to guide that understanding to include a broader perspective. Individual as well as collective psychological and social risk factors as well as symptoms should be identified alongside the somatic diagnostic process and included in a comprehensive physical–mental treatment plan on different levels. Therefore, the biopsychosocial model should be the underlying concept in both understanding and treating patients with Long-COVID syndrome. Since somatization is associated with a reduced ability to perceive and communicate affects, healthcare professionals should be open to recognizing, validating and discussing these affects with patients, even and especially if this is difficult or associated with resistance. Access to psychological treatment should not be limited to clinically diagnosed psychiatric illnesses, but also include patients with subclinical depressive, anxious or post-traumatic symptoms to assess possible correlations on an individual scale. Somatic treatment plans should be carefully evaluated concerning their potential benefits and risks equally and applied where indicated. Lastly, we strongly advise against treatment strategies of trying multiple biological treatments one after the other without including a biopsychosocial perspective, especially if these treatments (as is currently the case) lack sufficient scientific evidence.

## Data Availability

Enquiries about data availability should be directed to the authors.

## References

[CR1] Aberer E (2010). Epidemiologic, socioeconomic and psychosocial aspects in lupus erythematosus. Lupus.

[CR2] Anaya JM, Rojas M, Salinas ML, Rodríguez Y, Roa G, Lozano M, Rodríguez-Jiménez M, Montoya N, Zapata E, Monsalve DM, Acosta-Ampudia Y, Ramírez-Santana C (2021). 'Post-COVID syndrome. A case series and comprehensive review. Autoimmun Rev.

[CR3] Barker-Davies RM, O'Sullivan O, Senaratne KPP, Baker P, Cranley M, Dharm-Datta S, Ellis H, Goodall D, Gough M, Lewis S, Norman J, Papadopoulou T, Roscoe D, Sherwood D, Turner P, Walker T, Mistlin A, Phillip R, Nicol AM, Bennett AN, Bahadur S (2020). The Stanford Hall consensus statement for post-COVID-19 rehabilitation. Br J Sports Med.

[CR4] Chen PJ, Pusica Y, Sohaei D, Prassas I, Diamandis EP (2021). An overview of mental health during the COVID-19 pandemic. Diagnosis (berl).

[CR5] Chey WD, Kurlander J, Eswaran S (2015). Irritable bowel syndrome: a clinical review. JAMA.

[CR6] Crook H, Raza S, Nowell J, Young M, Edison P (2021). Long covid-mechanisms, risk factors, and management. BMJ.

[CR7] Engel GL (1978). The biopsychosocial model and the education of health professionals. Ann N Y Acad Sci.

[CR8] Fenton C, Lee A (2023). Antidepressants with anti-inflammatory properties may be useful in long COVID depression. Drugs Ther Perspect.

[CR9] Hare DL, Toukhsati SR, Johansson P, Jaarsma T (2014). Depression and cardiovascular disease: a clinical review. Eur Heart J.

[CR10] Harenwall S, Heywood-Everett S, Henderson R, Godsell S, Jordan S, Moore A, Philpot U, Shepherd K, Smith J, Bland AR (2021). Post-Covid-19 syndrome: improvements in health-related quality of life following psychology-led interdisciplinary virtual rehabilitation. J Prim Care Community Health.

[CR11] Harenwall S, S Heywood-Everett, R Henderson, J Smith, R McEnery, and AR Bland (2022) The interactive effects of post-traumatic stress symptoms and breathlessness on fatigue severity in Post-COVID-19 syndrome J Clin Med 11 10.3390/jcm11206214 PMC9604889 36294534

[CR12] Heitzman J (2020). Impact of COVID-19 pandemic on mental health. Psychiatr Pol.

[CR13] Henningsen P (2018). Management of somatic symptom disorder. Dialogues Clin Neurosci.

[CR14] Hossain MM, Tasnim S, Sultana A, Faizah F, Mazumder H, Zou L, McKyer ELJ, Ahmed HU, Ma P (2020). 'Epidemiology of mental health problems in COVID-19: a review. F1000Res.

[CR15] Joli J, Buck P, Zipfel S, Stengel A (2022). Post-COVID-19 fatigue: a systematic review. Front Psychiatry.

[CR16] Koczulla AR, T Ankermann, U Behrends, P Berlit, R Berner, S Böing, F Brinkmann, U Frank, C Franke, R Glöckl, C Gogoll, W Häuser, B Hohberger, G Huber, T Hummel, V Köllner, S Krause, J Kronsbein, T Maibaum, A Otto-Thöne, U Pecks, EMJ Peters, S Peters, M Pfeifer, T Platz, M Pletz, F Powitz, KF Rabe, C Scheibenbogen, D Schneider, A Stallmach, M Stegbauer, T Tenenbaum, N Töpfner, F von Versen-Höynck, HO Wagner, C Waller, CN Widmann, C Winterholler, H Wirtz, and R Zwick (2022) AWMF S1-Guideline Long/ Post-COVID. In. AWMF online: AWMF

[CR17] Kreipe RE (2006). The biopsychosocial approach to adolescents with somatoform disorders. Adolesc Med Clin.

[CR18] Lopez-Leon S, Wegman-Ostrosky T, Perelman C, Sepulveda R, Rebolledo PA, Cuapio A, Villapol S (2021). More than 50 long-term effects of COVID-19: a systematic review and meta-analysis. Sci Rep.

[CR19] Lund LC, Hallas J, Nielsen H, Koch A, Mogensen SH, Brun NC, Christiansen CF, Thomsen RW, Pottegård A (2021). Post-acute effects of SARS-CoV-2 infection in individuals not requiring hospital admission: a Danish population-based cohort study. Lancet Infect Dis.

[CR20] Manchia M, Gathier AW, Yapici-Eser H, Schmidt MV, de Quervain D, van Amelsvoort T, Bisson JI, Cryan JF, Howes OD, Pinto L, van der Wee NJ, Domschke K, Branchi I, Vinkers CH (2022). The impact of the prolonged COVID-19 pandemic on stress resilience and mental health: a critical review across waves. Eur Neuropsychopharmacol.

[CR21] Matta J, Wiernik E, Robineau O, Carrat F, Touvier M, Severi G, de Lamballerie X, Blanché H, Deleuze JF, Gouraud C, Hoertel N, Ranque B, Goldberg M, Zins M, Lemogne C (2022). Association of self-reported COVID-19 infection and SARS-CoV-2 serology test results with persistent physical symptoms among french adults during the COVID-19 pandemic. JAMA Intern Med.

[CR22] Mazza MG, Palladini M, Poletti S, Benedetti F (2022). Post-COVID-19 depressive symptoms: epidemiology, pathophysiology, and pharmacological treatment. CNS Drugs.

[CR23] Mentzos S (2017) [Textbook of psychodynamics: The function of mental disorder dysfunctionality] (Vandenhoeck and Ruprecht)

[CR24] Michaelides A, Zis P (2019). Depression, anxiety and acute pain: links and management challenges. Postgrad Med.

[CR25] Papadimitriou G (2017). The "biopsychosocial model": 40 years of application in psychiatry. Psychiatriki.

[CR26] Pinquart M, Duberstein PR (2010). Depression and cancer mortality: a meta-analysis. Psychol Med.

[CR27] Ringel Y, Drossman DA (2001). Psychosocial aspects of Crohn's disease. Surg Clin North Am.

[CR28] Silva Andrade B, S Siqueira, WR de Assis Soares, F de Souza Rangel, NO Santos, A Dos Santos Freitas, P Ribeiro da Silveira, S Tiwari, KJ Alzahrani, A Góes-Neto, V Azevedo, P Ghosh, and D Barh (2021) Long-COVID and Post-COVID health complications: An up-to-date review on clinical conditions and their possible molecular mechanisms. Viruses 13 10.3390/v13040700 PMC8072585 33919537

[CR29] Sivan M, Taylor S (2020). NICE guideline on long covid. BMJ.

[CR30] Tesarz J, Gerhardt A, Eich W (2018). Influence of early childhood stress exposure and traumatic life events on pain perception. Schmerz.

[CR31] Wainwright T, W and M Low (2020) Why the biopsychosocial model needs to be the underpinning philosophy in rehabilitation pathways for patients recovering from COVID-19. Integr Healthcare J 10.1136/ihj-2020-000043 PMC7474899 38607937

[CR32] Waller E, Scheidt CE (2006). Somatoform disorders as disorders of affect regulation: a development perspective. Int Rev Psychiatry.

[CR33] Yong SJ (2021). Long COVID or post-COVID-19 syndrome: putative pathophysiology, risk factors, and treatments. Infect Dis (lond).

